# Supplementary material for: Association between Pancreatoblastoma and Familial Adenomatous Polyposis: Review of the Literature with an Additional Case
Source: Genes (Basel). 2023 Dec 27;15(1):44. doi: 10.3390/genes15010044 (PMC10815143; doi:10.3390/genes15010044)
Supplement: Supplementary file 1 [file genes-15-00044-s001.zip › genes-2765709-supplementary.pdf]

Online Supplementary

Table S1: Detailed Search Strategy “PBL and FAP”.

**PubMed**<br>**(n= 9)**	("pancreatoblastoma"[MeSH Terms] OR "pancreatoblastoma"[All Fields] OR "PBL"[All Fields]) AND ("familial adenomatous polyposis"[All Fields] OR "adenomatous polyposis coli"[All Fields] OR "FAP"[All Fields])
**Ovid**<br>**(n=15)**	**1** PBL.mp. [mp=ti, ab, hw, tn, ot, dm, mf, dv, kf, fx, dq, bt, nm, ox, px, rx, an, ui, sy, ux, mx] (21860)<br>**2** pancreatoblastoma.mp. [mp=ti, ab, hw, tn, ot, dm, mf, dv, kf, fx, dq, bt, nm, ox, px, rx, an, ui, sy, ux, mx] (832)<br>**3** familial adenomatous polyposis coli.mp. [mp=ti, ab, hw, tn, ot, dm, mf, dv, kf, fx, dq, bt, nm, ox, px, rx, an, ui, sy, ux, mx] (414)<br>**4** FAP.mp. [mp=ti, ab, hw, tn, ot, dm, mf, dv, kf, fx, dq, bt, nm, ox, px, rx, an, ui, sy, ux, mx] (17250)<br>**5** Adenomatous polyposis coli.mp. [mp=ti, ab, hw, tn, ot, dm, mf, dv, kf, fx, dq, bt, nm, ox, px, rx, an, ui, sy, ux, mx] (13874)<br>**6** familial adenomatous polyposis.mp. [mp=ti, ab, hw, tn, ot, dm, mf, dv, kf, fx, dq, bt, nm, ox, px, rx, an, ui, sy, ux, mx] (13612)<br>**7** 1 or 2 (22667)<br>**8** 3 or 4 or 5 or 6 (33126)<br>**9** 7 and 8 (22)<br>**10** limit 9 to english language (22)<br>**11** limit 10 to human (21)<br>**12** remove duplicates from 11 (15)
**Scopus**<br>**(n=2)**	('pancreatoblastoma' OR 'pbl') AND ('familial adenomatous polyposis' OR 'fap' OR 'adenomatous polyposis coli' OR 'familial adenomatous polyposis coli')

Table S2: Detailed Search Strategy “PBL and adult”.

**PubMed (n= 63)**	("pancreatoblastoma"[MeSH Terms] OR "pancreatoblastoma"[All Fields] OR "PBL"[All Fields]) AND ("adult"[All Fields])
**Ovid (n=180)**	**1** pancreatoblastoma.mp. [mp=ti, ot, ab, tx, kw, ct, sh, hw, tn, dm, mf, dv, kf, fx, dq, bt, nm, ox, px, rx, an, ui, sy, ux, mx] (837)<br>**2** adult.mp. [mp=ti, ot, ab, tx, kw, ct, sh, hw, tn, dm, mf, dv, kf, fx, dq, bt, nm, ox, px, rx, an, ui, sy, ux, mx] (16131418)<br>**3** 1 and 2 (281)<br>**4** limit 3 to english language [Limit not valid in CDSR; records were retained] (267)<br>**5** limit 4 to human [Limit not valid in CDSR; records were retained] (259)<br>**6** remove duplicates from 5 (180)
**Scopus (n=166)**	('pancreatoblastoma' OR 'pbl') AND (adult')

Fig.S1 PRISMA flowchart of study selection “PBL and adult”

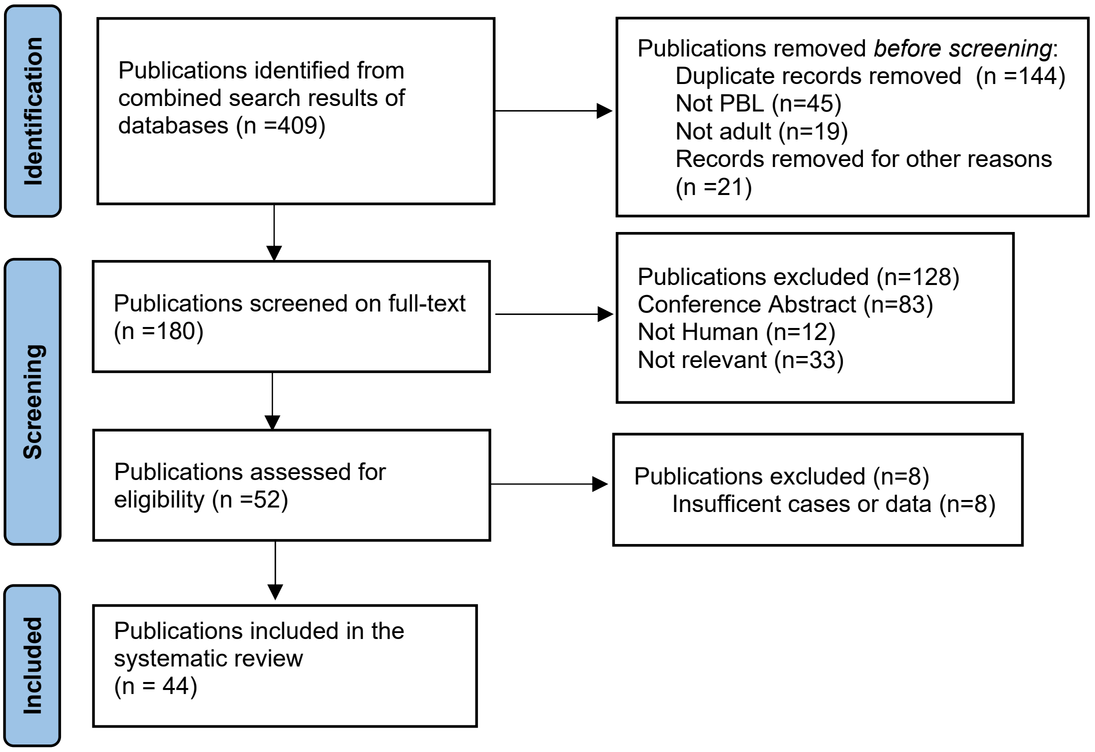

Table S3: Study characteristics "PBL and adult"

Study, y	Age diagnosed,y	Sex, F/M	Size, cm
Palosaari et al. 1986	37	M	8
Hoorens et al. 1994	39	F	13
Klimstra et al.1995	19,36,37,54,56	4 M, 1 F	15,12,20,20,9
Dunn and Longnecker. 1995	61	M	9
Hayasaki *et al. 1999*	48	F	5
Montemarano et al.2000	20	F	na
Robin et al. 1997	20	M	9
Levey and Banner, 1996	68	F	9
Mumme *et al. 2001*	22	F	9
Benoist *et al. 2001*	48	F	10
Gruppioni *et al. 2002*	30	M	8
Du *et al. 2003*	78	F	2.7
Pitman and Faquin .2004	18	M	9
Rosebrook *et al.*2005	29	F	2
Sheng *et al.*2005	18	M	10
Zhu *et al.* 2005	24	F	4
Kuxhaus *et al.* 2005	69	M	na
Rajpal *et al.*2006	50	M	13
Charlton-Ouw et al. 2008	33	M	5
Ohike *et al. 2008*	74	F	4.5
Cavallini *et al.*2009	69,26	2M	6,5
Comper *et al.*2009	27,69	2M	5.5, 5.5
Savastano *et al.* 2009	36	F	4.3
Boix et al. 2010	33	F	3.5
Balasundaram et al.2012	27	F	3.6
Gringeri *et al.*2012	38	F	na
Hammer and Owens. N2013	37	M	7
Redelman *et al. 2014*	26	F	7
Salman *et al. 2013*	60, 51, 58	3M	1.8, 4 , 4.5
Efstratios Z. 2015	24	M	8
Marisa T. 2016	34	M	5.8
Chen *et al.* 2018	26	M	6.7
Nunes *et al.* 2018	31	M	4
Vilaverde *et al.* 2016	37	F	17
Zouros *et al.* 2015	24	M	8
Kuxhaus *et al.* 2005	69	M	na
Liu *et al.* 2020	49	M	na
Morrissey et *al.* 2020	69	F	5.5
Zhang *et al.*2020	55, 37, 36, 37, 22, 65, 61	7M	4.3, 4.7, 5.5, 6.6, 12.4, 5, 5.6

Elghawy *et al.* 2021	23	F	na
Slack JC et al. 2022	57	M	4.8
Snyder *et al.* 2020	28	F	na
Wu M et al. 2022	26,47,64,68,69,65,58	5F, 2 M	6.6, 6.4, 3.7, 4.6, 6.4, 6.2, 3.5
Kulak O. 2023	80, 81	1M, 2F	5.4, 2.7
